# Inherent Bias in Artificial Intelligence-Based Decision Support Systems for Healthcare

**DOI:** 10.3390/medicina56030141

**Published:** 2020-03-20

**Authors:** Varadraj Gurupur, Thomas T. H. Wan

**Affiliations:** Department of Health Management and Informatics, University of Central Florida, Orlando, FL 32816, USA; thomas.wan@ucf.edu

**Keywords:** knowledge bias, decision support systems, artificial intelligence, healthcare information systems, knowledge-based systems

## Abstract

The objective of this article is to discuss the inherent bias involved with artificial intelligence-based decision support systems for healthcare. In this article, the authors describe some relevant work published in this area. A proposed overview of solutions is also presented. The authors believe that the information presented in this article will enhance the readers’ understanding of this inherent bias and add to the discussion on this topic. Finally, the authors discuss an overview of the need to implement transdisciplinary solutions that can be used to mitigate this bias.

## 1. Understanding the Concept of Bias

Available literature indicates that artificial intelligence-based systems used in healthcare have flaws that adversely affect its ability to perform at an expected level [1]. This is mainly due to the presence of an inherent bias associated with these systems. Thereby, establishing a critical need to comprehend a few key concepts associated with this bias. For the purpose of analyzing this bias, it is important to perceive an artificial intelligence-based system as an information system. Therefore, it is important to analyze a few key concepts associated with information bias.

Althubaiti [2] defines information bias in healthcare as “any systematic error in the design, conduct, or analysis of a study.” This sheds light on two important types of bias: (a) Information bias, and (b) selection bias. With respect to the bias from experimental design, Althubaiti [2] argues that many times this form of bias is unintentional in nature. In this literature, the author also presents the idea that self-reporting systems may be biased due to the sampling approach (especially when using convenience sampling), recall period, and selective recall.

It is important to note that an artificial intelligence-based decision support system uses knowledge derived from available literature and other available forms of experimental results. Under these circumstances, the bias in discussion can be conceived as an outcome of the methods and its associated selection processes used for experimentation. On this matter, Gurupur et al. [3] have explained how methods used for analysis can affect the accuracy of the outcome. In their experiment the investigators have provided a greater emphasis on computationally stronger techniques that consume more computational power for analyzing healthcare data. It is worth noting that accuracy of analysis is also dependent on the accuracy of the input data used for this purpose. Based on this argument we now have three essential parameters that lead to an overall bias in the knowledge base of a recommendation system: (a) Bias due to inaccurate data analysis, (b) bias occurring due to false information derived from a credible resource, and (c) bias occurring due to experimental design and implementation. 

Data and knowledge engineers involved in the synthesis of decision support systems must take into consideration these categories of bias while developing the system [4]. Henriksen and Kaplan [5] emphasize on how hindsight bias affects the healthcare delivery system. They define hindsight bias as “the tendency for people with outcome knowledge to exaggerate the extent to which they would have predicted the event beforehand.” Additionally, they also identify outcome bias being correlated with the influence of outcome knowledge acquired by the investigator/s. Hindsight bias is an important concept that cannot be obviated in a discussion that involves artificial intelligence-based systems for healthcare.

## 2. Information Explosion and the Need for Reliability

In the last decade, scientists working with the healthcare industry involved in synthesis, design, and development of decision support systems have witnessed an exponential increase in the application of artificial intelligence. This surge is partly based on the prevalence of information explosion and its possible use in developing information systems. The phenomenon of information explosion has been described by Edward Huth in [6]. In this work, Huth supports the idea of information explosion based on the growth of information published with the US National Library of Medicine. Huth perceives this phenomenon in terms of the growth in medical science and its associated concepts. On a similar context, Tony Dixon [7] describes the dilemmas in dealing with information explosion for physicians. Here, Dixon differentiates between solicitated and unsolicited work while providing emphasis on solicitated work. Although this article was published prior to the advent of World Wide Web, it sheds some light on a very important concept of reliable information. It is important to note that reliable information is critically connected with the idea of making accurate predictions. 

To further explore the need for reliability, Vrij et al. [8] have explained the use of reliable information in investigative interviews. Here, the investigators correlate the concept of reliability to the idea of using the right process for gathering information. Although it is possible to argue that the authors are digressing the discussion towards a different domain of science, it is important to identify the fact that artificial intelligence-based systems attempt to mimic human cognition and thinking. Literature suggests that the availability of reliable source of information results should be considered before and during the design of an artificial intelligence-based system. An artificial intelligence-based system that is built on unreliable sources results in poor reliability of the system. The authors are of the opinion that the information source needs to be a criterion during the implementation of the system. In this context, John Bohannon in his article [9] warns the investigators of the possibility of unreliable information being published in reputable journals. This very interesting work delineates a sting operation conducted by him by sending false journal articles to many journals. This established the idea that name recognition of the source sometimes cannot guarantee reliable information. In the same context, Roberts et al. [10] debated on the idea of review articles being a reliable source for biological conservation. 

This discussion is further explored by Fragale and Heath [11]. In their work, they correlate between credible sources and believable facts. The authors here hypothesize the idea of belief force equaling credible source in terms of human psychology. Thereby, leading to indicate the fact that source credibility influences belief even when the information received is false. If this theory is true it further exacerbates the possibility of information bias, and inaccurate and false predictions. Here, we are essentially dealing with two terms: Precision and accuracy. Precision is defined by Cambridge dictionary as [12] “the level of agreement of a particular measurement with itself when it is repeated.” For an artificial intelligence-based decision support system [13] to provide the right recommendation there must be a greater emphasis on accuracy when compared to precision. This is mainly because precision in theory may lead to repeated wrong recommendations while the improvement in learning from new knowledge must essentially lead to right recommendations that ties it to the concept of accuracy. In this discussion, it is also important to note that Cambridge dictionary defines accuracy as, [14] “the agreement of a particular measurement with an accepted standard.” 

## 3. Problems in Processing Knowledge

So far, a discussion of bias in data and knowledge has been presented. However, it is important to note that the algorithm processing the biased knowledge may be biased, as well. Nelson [15] states that, “bias is a reflection of the data algorithm authors choose to use.” He also connects the concept of bias to perceived fairness where a negligible level of bias is considered allowable. Additionally, the literature also delineates the idea that electronic health record data can be shallower for segments of the population based on how the data is curated and distributed by brokers. Here, it is to be noted that most of the recommendation engines work on data captured by different data vendors. The problem with this approach is that it might lead to a domino effect further amplifying bias and adversely affecting healthcare outcomes; thereby, leading to catastrophic results such as death of a patient. Therefore, it is important to use decision support systems for recommendation purposes only and not for having them perform automated diagnostic procedures. If we must take into account the idea that domain knowledge is curated into the system by a domain knowledge expert, as illustrated by Gurupur et al. [16], the inference performed by the recommendation engine will be at the mercy of the biases and other limitations of knowledge acquired by the domain expert. 

Considering such systems for healthcare, the domain experts can be broadly categorized into practicing physicians, nurse practitioners, physician assistants, and health information managers. This leads us to the concept of hindsight discussed by Henriksen and Kaplan [5]. Interestingly, the concept of hindsight also connects with the idea of a reliable source of information [8]. This leads us to question the level of expertise acquired by the domain knowledge experts [17] responsible for knowledge curation. In other words, is the physician or the nurse practitioner at task a reliable source of information? To further explore this idea, Wan [18] has described the use of explicit knowledge learned from the literature or documents, tacit knowledge gained from personal experiences, and implicit knowledge acquired from the application of explicit knowledge that can be used to develop information systems for healthcare. Shallow or superficial knowledge in any of these three categories of knowledge and poorly defined problems in knowledge acquisition processes, especially in the case of a self-learning system, may result in unexpected bias.

## 4. Possible Solutions

In summary, we can now broadly categorize reasons for bias in decision support systems using artificial intelligence for healthcare broadly into two categories: a) Bias in knowledge captured, and b) problems in efficiently processing knowledge acquired. Table 1 provides an illustration of the summary of this knowledge bias. Additionally, Table 2 provides a summary of bias involved in processing knowledge.

Interestingly, Dressel and Farid [19] have reported that a widely used tool for prediction to predict recidivism was only as accurate as human judgement. A plausible method of measuring reliability or completeness of information could be ascertaining information gain [20] using information entropy. Here, we are attempting to measure the integrity of the knowledge and the level of bias of a decision support system for healthcare by measuring the probability of an accurate outcome. Incidentally, information entropy has also been used by Gurupur et al. [21] to measure the strength of a knowledge base built using concept maps. 

This essentially means that when our analysis and associated measurements of those analysis is inherently biased there is a need to develop another (higher) level of measurement to ascertain the quality or level of bias involved in the lower level of measurement. From this perspective, given the problem of inherent bias in analysis, knowledge, and algorithms an overall systems analysis of measurements is a much-needed utility to ascertain the strength of an artificial intelligence-based decision support system. The core purpose of this measurement must be conceived as an attempt to mitigate the inherent bias manifesting in different shapes and forms as discussed in this article.

## 5. Conclusions

The facts and arguments presented in this article clearly indicate that a further validation of the reliability, validity, and applicability of an artificial intelligence-based decision support system for healthcare is required under a theoretically informed framework. For instance, a transdisciplinary perspective advocates healthcare outcomes or health status improvement as a joint function of both micro-level and macro-level predictors [22]. The determinants of population health are then classified into person-level, as well as ecological-level factors. Thus, the interplay between the two-level predictors is an important area of causal inquiries. Ultimately, researchers are expected to employ both deductive and inductive logics for their scientific pursuit. With no exception, scientists involved in developing clinical and administrative decision support systems for healthcare should perform a confirmatory approach in validating the integrity of theoretical formulation and empirical validation. This leads to a critical concept of “measurement rigor of measurements” that is much needed to mitigate the inherent bias in these systems. Additionally, there is a critical need for transformative transdisciplinary approaches to mitigate this bias. Based on this discussion, the authors establish a need to develop metrics for measurement rigors in artificial intelligence-based decision support systems for healthcare. The authors intend to base their future research work on fulfilling this need.

## Figures and Tables

**Table 1:** Important factors leading to knowledge bias.

Factors Leading to Knowledge Bias	Description
Experimental bias	Inherent bias in experiment leading to inaccurate outcomes, and pre-existing beliefs leading to wrong perceptions such as hindsight
Problems with information reliability	Synthesizing systems based on false or partially accurate data
Limited expert knowledge	Domain experts may have limited knowledge of their own domain that will limit the knowledge programmed into the system
Shallow information	Implicit knowledge contained in systems such as electronic health records may be shallow and may not include the necessary details

**Table 2:** Important factors leading to processing bias.

Factors Leading to Processing Bias	Description
Bias in the selected algorithm	The selected data processing algorithm may not be appropriate for the required decision support process
Bias in tacit knowledge used for feedback	Feedback provided by knowledge providers may be biased and may create bias if used to modify the processing structure
